# Supplementary material for: Meta-analytic connectivity modelling of deception-related brain regions
Source: PLoS One. 2021 Aug 25;16(8):e0248909. doi: 10.1371/journal.pone.0248909 (PMC8386837; doi:10.1371/journal.pone.0248909)
Supplement: S1 Fig — The uncorrected estimate of meta-analytic connectivity between each seed region and all other specified nodes. (PDF) [file pone.0248909.s001.pdf]

	Projection ALE Values:						
**Seeds:**	**L Ins**	**L SFG**	**R Ins**	**R SMG**	**L SMG**	**L MFG**	**R MFG**
**L Ins**		0	0	0.11735	0.07968	0.5865	0
**L SFG**	0		0	0.03743	0.17955	0	0
**R Ins**	0	0		0	0.02686	0.13334	0
**R SMG**	0.00021	0.00001	0		0.00015	0.00305	0.00013
**L SMG**	0	0.00016	0.00002	0		0.0046	0.00001
**L MFG**	0.01184	0	0.00881	0.00348	0.05574		0.00825
**R MFG**	0	0	0	0.00058	0.01057	0.03082	
