# Supplementary figures and images for: Meta-analytic connectivity modelling of deception-related brain regions
Source: PLoS One. 2021 Aug 25;16(8):e0248909. doi: 10.1371/journal.pone.0248909 (PMC8386837; doi:10.1371/journal.pone.0248909)

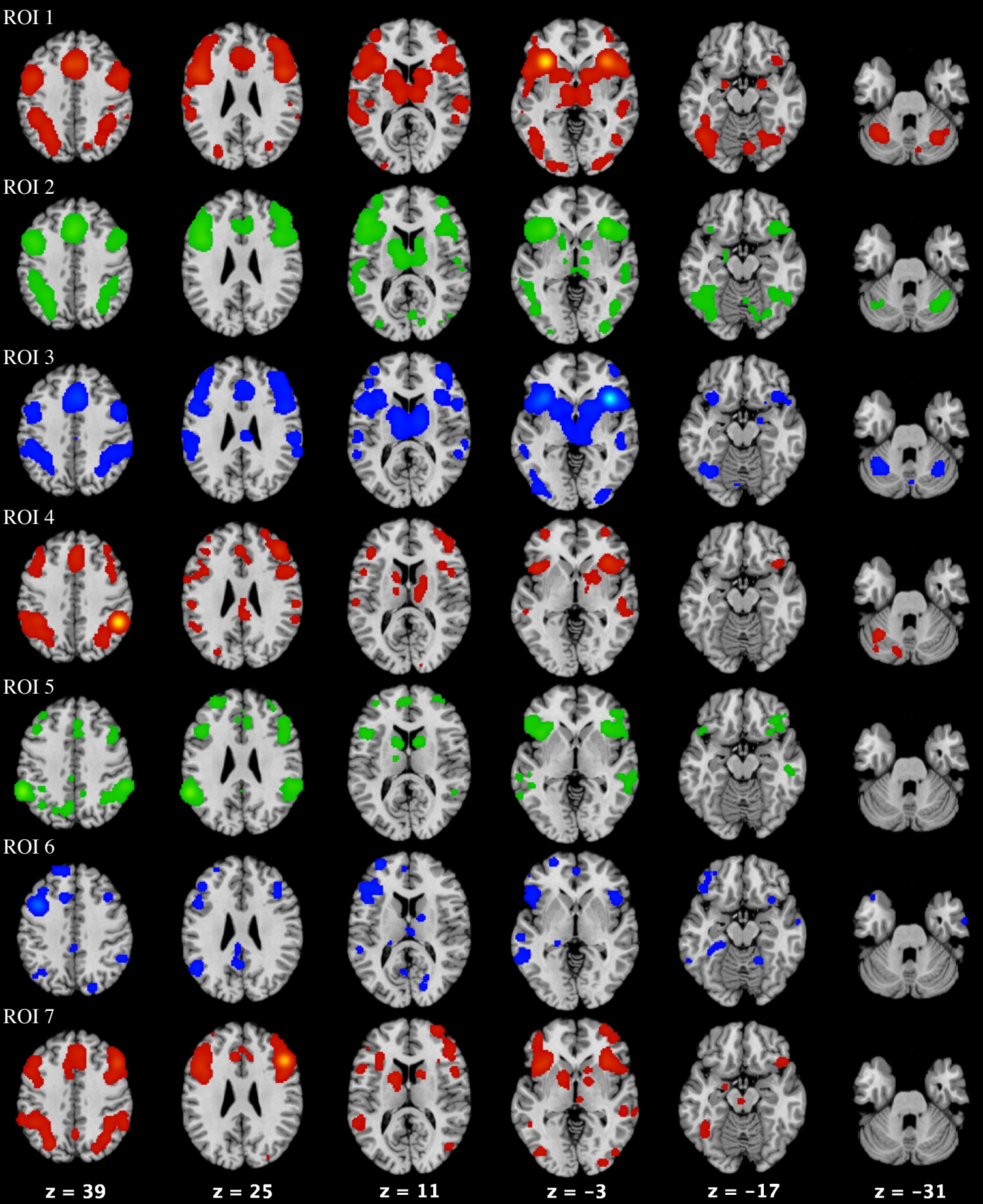

Supplement: S2 Fig — Each of the 7 thresholded ALE maps representative of each MACM. Six horizontal slices (z = 39, 25, 11, -3, -17, and -31) are shown for each ROI (1: left insula, 2: left superior frontal gyrus, 3: right insula, 4: right supramarginal gyrus, 5: left supramarginal gyrus, 6: left medial frontal gyrus, 7: right medial frontal gyrus). (PDF) [file pone.0248909.s002.pdf]

A

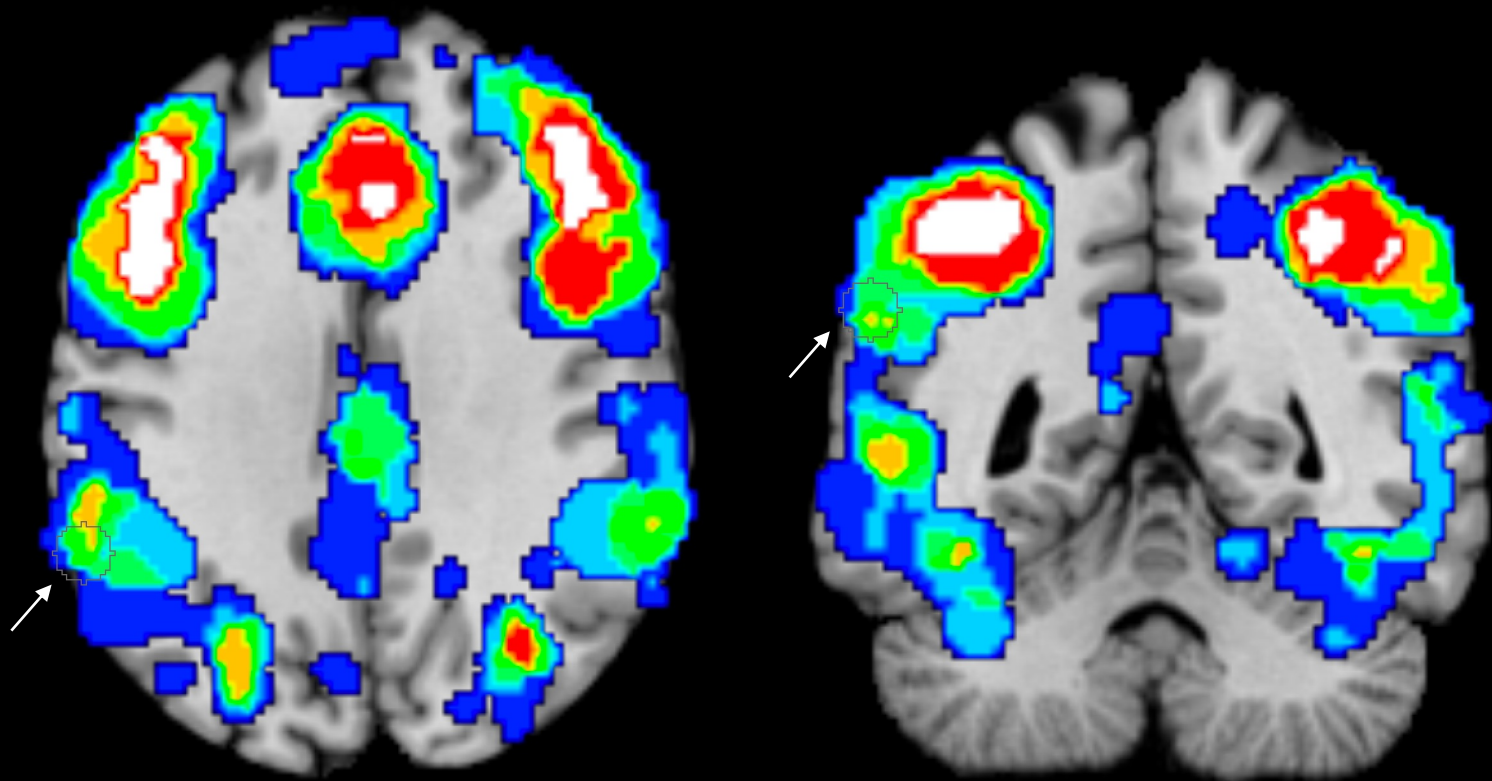

B

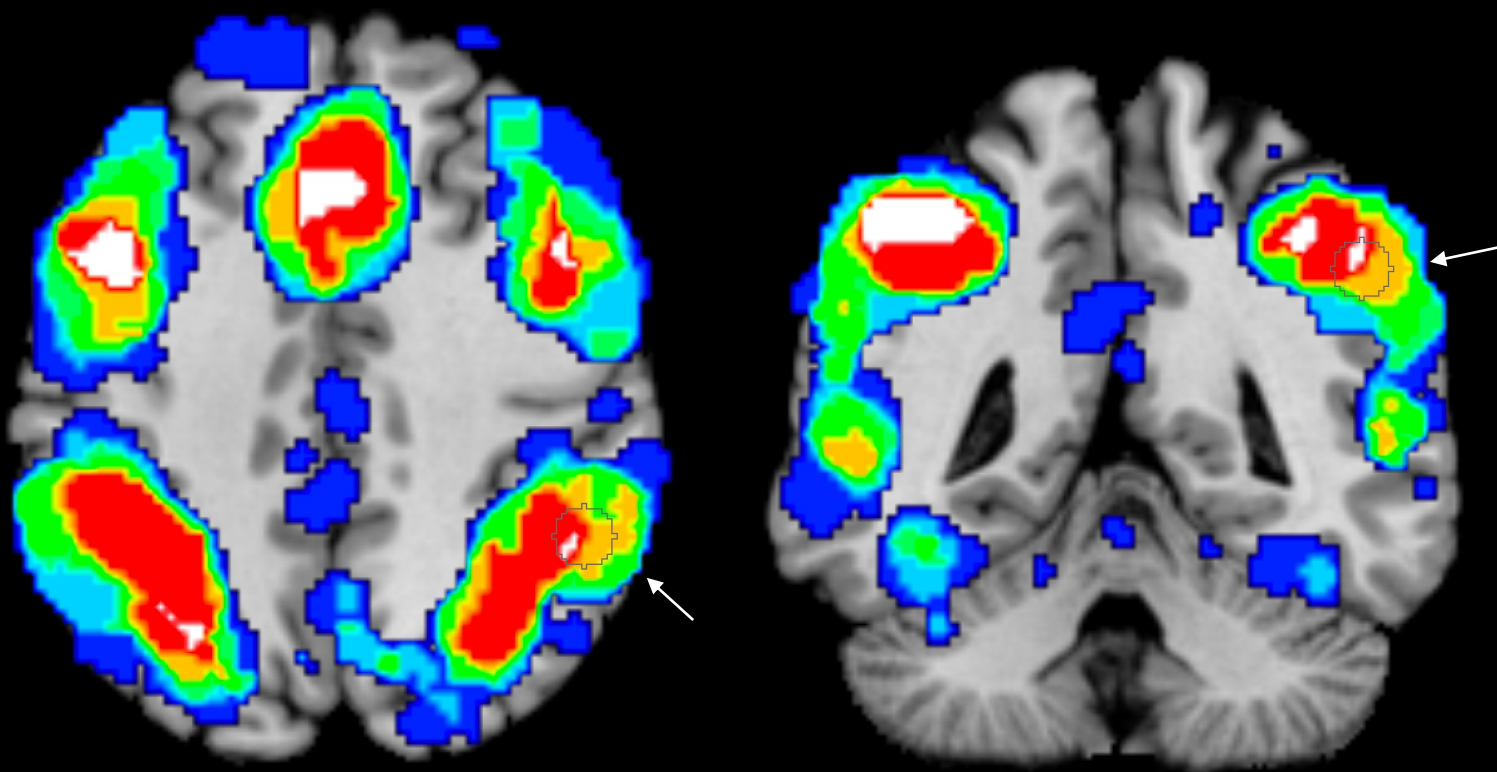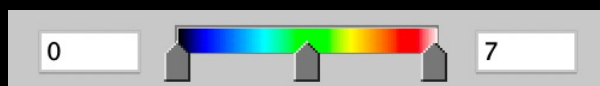

Supplement: S3 Fig — The 7 P value maps overlaid onto a template brain demonstrate convergence at the right (A) and left (B) SMG (supramarginal gyrus) ROIs, which are indicated by gray circles and white arrows. Horizontal slice is on the left, and coronal slice is on the right. (PDF) [file pone.0248909.s003.pdf]
