# Supplementary material for: Meta-analytic connectivity modelling of deception-related brain regions
Source: PLoS One. 2021 Aug 25;16(8):e0248909. doi: 10.1371/journal.pone.0248909 (PMC8386837; doi:10.1371/journal.pone.0248909)
Supplement: S1 Table — This ALE consisted of 46 studies and 202 experiments with 1,423 foci from 4,678 participants. (DOCX) [file pone.0248909.s004.docx]

| # | Reference | N | Foci | Contrast Reported | Deception Task |
| --- | --- | --- | --- | --- | --- |
| 1 | Abe et al., 2014 | 25 | 10 | Dishonest + Honest > Control | Decision-making (harmful or helpful) |
|  |  |  | 4 | (Dishonest/Harmful + Honest/Harmful) > (Dishonest/Helpful + Honest/Helpful) |  |
|  |  |  | 3 | (Dishonest/Helpful + Honest/Helpful) > (Dishonest/Harmful + Honest/Harmful) |  |
|  |  |  | 3 | Dishonest/Harmful > Honest/Harmful |  |
|  |  |  | 5 | (Honest/Harmful + Honest/Helpful) > (Dishonest/Harmful + Dishonest/Helpful) |  |
|  |  |  | 4 | Honest/Harmful > Dishonest/Harmful |  |
| 2 | Abe & Greene, 2014 | 13 | 3 | Honest: Opportunity Loss > No-Opportunity Loss | Monetary Incentive Delay/Incentive Prediction |
|  |  | 8 | 1 | Dishonest: Opportunity Win > No-Opportunity Win |  |
|  |  |  | 3 | Dishonest: Opportunity Loss > No-Opportunity Loss |  |
|  |  | 7 | 1 | Ambiguous & Dishonest: Opportunity Win > No-Opportunity Win |  |
|  |  |  | 7 | Ambiguous & Dishonest: Opportunity Loss > No-Opportunity Loss |  |
|  |  | 7 | 4 | Ambiguous: Opportunity Loss > No-Opportunity Loss |  |
| 3 | Baumgartner et al., 2009 | 26 | 1 | Promise Stage: Dishonest > Honest, (Promise - No Promise)^Dishonest - Promise - No Promise)^Honest | Modified Economic Trust Game |
|  |  |  | 1 | Anticipation Stage: Dishonest > Honest, (Promise - No Promise)^Dishonest - (Promise - No Promise)^Honest |  |
|  |  |  | 1 | Anticipation Stage: Dishonest > Honest, (Promise - No Promise)^Honest - (Promise - No Promise)^Dishonest |  |
|  |  |  | 1 | Anticipation Stage: Dishonest > Honest, ((No Promise - Promise)^Dishonest - (No Promise - Promise)^Honest), p<0.0001 |  |
|  |  |  | 1 | Anticipation Stage: Dishonest > Honest, ((No Promise - Promise)^Dishonest - (No Promise - Promise)^Honest), p<0.0005 |  |
|  |  |  | 2 | Anticipation Stage: Dishonest > Honest, ((No Promise - Promise)^Dishonest - (No Promise - Promise)^Honest), p<0.005 |  |
|  |  |  | 2 | Decision Stage A: Dishonest > Honest, ((Promise - No Promise)^Dishonest - (Promise - No Promise)^Honest), p<0.001 |  |
|  |  |  | 2 | Decision Stage A: Dishonest > Honest, ((Promise - No Promise)^Dishonest - (Promise - No Promise)^Honest), p<0.005 |  |
|  |  |  | 1 | Decision Stage B: Dishonest > Honest, (Promise - No Promise)^Dishonest - (Promise - No Promise)^Honest |  |
| 4 | Bereczkei et al., 2015 | 16 | 2 | Unfair - Control, High Machiavellian > Low Machiavellian | Trust Game (in fair or unfair situations) |
|  |  |  | 7 | Fair - Control, High Machiavellian > Low Machiavellian |  |
|  |  | 38 | 15 | Fair > Control |  |
|  |  |  | 9 | Unfair > Control |  |
| 5 | Bhatt et al., 2009 | 18 | 9 | Unfamiliar: Lie > Truth | Recognition/"Line-up" |
|  |  |  | 4 | Familiar: Lie > Truth |  |
|  |  |  | 4 | Familiar (Lie > Truth) > Unfamiliar (Lie > Truth) |  |
| 6 | Browndyke et al., 2008 | 7 | 7 | Malingered Recognition Miss > Normal Recognition Hits | Recognition Memory/Feigned Memory Impairment |
|  |  |  | 5 | Malingered Recognition False Alarm Errors > Normal Recognition Correct Rejection |  |
|  |  |  | 4 | Normal Hits > Malingered Target Miss |  |
|  |  |  | 5 | Normal Correct Rejection > Malingered False Alarm |  |
| 7 | Cui et al., 2014 | 16 | 8 | Assigned Murderer Group: Deceptive Probe Answer Judged Truthful > Truthful Irrelevant Answer Judged Truthful | Mock Murder/Modified Guilty Knowledge Test |
|  |  |  | 12 | Deceptive Probe Answer Judged Truthful: Assigned Murderer Group > Assigned Innocent Group |  |
|  |  |  | 7 | Probe: Assigned Murderer Group > Assigned Innocent Group |  |
|  |  |  | 4 | Assigned Murderer Group: Probe > Irrelevant |  |
| 8 | Ding et al., 2012 | 12 | 7 | Identity Concealment > Control | Recognition/Identity Concealment |
|  |  |  | 9 | Identity Faking > Control |  |
| 9 | Farrow et al., 2015 | 20 | 5 | Impression-Management > Control | "Balanced Inventory of Desirable Responding" |
|  |  |  | 2 | Self-deception > Control |  |
|  |  |  | 7 | Faking bad > Control |  |
|  |  |  | 7 | Impression-Management Main Effects |  |
|  |  |  | 2 | Self-Deception Main Effects |  |
|  |  |  | 29 | (Impression-Management Faking bad & Self-Deception Faking Good[+1])  vs. (Impression-Management Faking Good & Self-Deception Faking Bad[-1]) Main Effects |  |
|  |  |  | 14 | Faking Bad Main Effects |  |
| 10 | Fullam et al., 2009 | 24 | 2 | Lie - Truth | Lying (about performing tasks) |
|  |  |  | 4 | Truth - Lie |  |
| 11 | Greene & Paxton, 2009 | 14 | 2 | Dishonest (Opportunity Win > No-Opportunity Win) | Computerized Coin Flips/Moral Judgement |
|  |  |  | 7 | Dishonest (Opportunity Loss > No-Opportunity Loss) |  |
|  |  |  | 2 | Honest (Opportunity Win > No-Opportunity Win) |  |
|  |  |  | 3 | Honest (Opportunity Win > Opportunity Loss) |  |
| 12 | Harada et al., 2009 | 18 | 23 | Lie Judgement - Gender Judgment (masked with Lie Judgement) | Control Gender Judgement/Moral Judgement/Lie Judgement |
|  |  |  | 7 | Lie Judgement - Moral Judgement (masked with Lie Judgement) |  |
|  |  |  | 9 | Moral Judgement - Gender Judgment (masked with Moral Judgement) |  |
| 13 | Hayashi et al., 2014 | 37 | 6 | Harmful/ Dishonest > Harmful/ Honest | Harmful or Helpful Story-telling |
|  |  |  | 3 | Helpful/ Dishonest > Helpful/ Honest |  |
|  |  |  | 3 | Harmful/ Honest > Harmful/ Dishonest |  |
|  |  |  | 12 | Helpful/ Honest > Helpful/ Dishonest |  |
| 14 | Ito et al., 2011 | 32 | 9 | Main effect of 'Lie' (Neutral/Lie+Negative/Lie) > (Neutral/Truth+Negative/Truth) | Remembering Neutral and Emotional Events |
|  |  |  | 8 | Neutral/Lie > Neutral/Truth |  |
|  |  |  | 8 | Negative/Lie > Negative/Truth |  |
|  |  |  | 5 | Conjunction Analysis: Neutral/Lie > Neutral/Truth + Negative/Lie > Negative/Truth |  |
|  |  |  | 7 | Main Effect of 'Negative' (Negative/Lie + Negative/Truth) > (Neutral/Lie + Neutral/Truth) |  |
|  |  |  | 3 | Main Effect of 'Neutral' (Neutral/Lie + Neutral/Truth) > (Negative/Lie + Negative/Truth) |  |
| 15 | Ito et al., 2012 | 16 | 6 | Execution: (Certain/Lie + Uncertain/Lie) > (Certain/Truth + Uncertain/Truth) | Modified Recognition Memory |
|  |  |  | 1 | Execution: (Uncertain/Truth + Uncertain/Lie) > (Certain/Truth + Certain/Lie) |  |
|  |  |  | 5 | Preparation: (Certain/Truth Cue + Certain/Lie Cue) > Uncertain Cue |  |
| 16 | Jiang et al., 2015 | 32 | 19 | Lie > True | Strategy Devising |
|  |  |  | 10 | True > Lie |  |
| 17 | Kireev et al., 2013 | 36 | 27 | (Conjunction) Deceptive Claim > Catch + Honest Claim > Catch | "Cheat" Card Game |
|  |  |  | 19 | Deceptive Claim > Catch |  |
|  |  |  | 21 | Deception Claim > Honest Claim |  |
|  |  |  | 6 | rCBF: Deceptive Claim > Catch |  |
|  |  |  | 17 | Honest Claim > Catch |  |
|  |  |  | 3 | Catch > Deceptive Claim |  |
| 18 | Kozel et al., 2005 | 30 | 18 | Lie - Truth, Model Building Group | Mock Crime/"Ring-Watch Testing" |
|  |  | 31 | 14 | Lie - Truth, Model Testing Group |  |
| 19 | Kozel et al., 2009 | 22 | 30 | Mock-Crime: Lie > True | Mock Crime/"Ring-Watch Testing" |
|  |  | 26 | 15 | No-Crime: Lie > True |  |
| 20 | Langleben et al., 2005 | 26 | 19 | Lie > Repeat Distracter | Modified Guilty Knowledge Test |
|  |  |  | 4 | Lie > Truth |  |
|  |  |  | 39 | Truth > Lie |  |
| 21 | Lee et al., 2009 | 10 | 8 | Intentional Faked Responses > Truthful Accurate Responses | Recognition/Feigned Memory Impairment |
|  |  |  | 3 | Intentional Faked Responses > Truthful Error Responses |  |
|  |  |  | 1 | Truthful Accurate Responses > Intentional Faked Responses |  |
| 22 | Lee et al., 2010 | 14 | 11 | Lie > True | Lying (about valence of pictures) |
|  |  |  | 17 | Positive: Lie > True |  |
|  |  |  | 4 | Negative: Lie > True |  |
|  |  |  | 4 | Conjunction Analysis (Lie > True, Positive + Negative) |  |
|  |  |  | 6 | True > Lie |  |
|  |  |  | 12 | Lie: Positive > Negative |  |
|  |  |  | 9 | Lie: Negative > Positive |  |
|  |  |  | 6 | True: Positive > Negative |  |
|  |  |  | 17 | True: Negative > Positive |  |
| 23 | Lee et al., 2013 | 13 | 2 | Main effect of Cue, Lie > Truth | Facial Recognition |
|  |  |  | 1 | Main Effect of Familiarity, Familiar > Unfamiliar |  |
| 24 | Lelieveld et al., 2016 | 44 | 6 | Justifiable Lies > Honest Reports | Evaluating Lies of Others |
|  |  |  | 6 | Unjustifiable Lies > Honest Reports |  |
|  |  |  | 6 | Justifiable Lies > Unjustifiable Lies |  |
|  |  |  | 2 | (Justifiable Lies > Unjustifiable Lies) Large Deviance > (Justifiable Lies > Unjustifiable Lies) Small Deviance |  |
|  |  |  | 5 | Justifiable Lies > Unjustifiable Lies for Large Deviance |  |
| 25 | Lissek et al., 2008 | 13 | 19 | Deception > Cooperation | Theory of Mind Task |
|  |  |  | 13 | Deception > Cooperation/Deception |  |
|  |  |  | 15 | Cooperation/Deception > Cooperation |  |
|  |  |  | 16 | Cooperation/Deception > Deception |  |
|  |  |  | 12 | Cooperation > Deception |  |
|  |  |  | 8 | Cooperation > Cooperation/Deception |  |
| 26 | Liu et al., 2012 | 14 | 16 | Falsification Card > BL | Conditional Proposition Testing |
|  |  |  | 9 | Falsification > Non-Falsification |  |
|  |  |  | 9 | Non-Falsification Card > BL |  |
|  |  |  | 14 | MT > Non-Falsification |  |
| 27 | Marchewka et al., 2012 | 29 | 13 | Lie > Truth (General + Personal) | Gender Identity Inventory |
|  |  |  | 13 | Lie > Truth (General) |  |
|  |  |  | 15 | Lie > Truth (Personal) |  |
|  |  | 14 | 16 | Males: Lie > Truth |  |
|  |  | 15 | 9 | Females: Lie > Truth |  |
|  |  | 14 | 11 | Males: General Lie > General Truth |  |
|  |  | 15 | 11 | Females: General Lie > General Truth |  |
|  |  |  | 13 | Males: Personal Lie > Personal Truth |  |
|  |  |  | 3 | Females: Personal Lie > Personal Truth |  |
|  |  |  | 9 | Personal Lie > General Lie |  |
| 28 | McPherson et al., 2012 | 15 | 8 | Tones: Feigned > Correct | Feigned Hearing Loss |
|  |  |  | 8 | Tones: Feigned > Incorrect |  |
|  |  |  | 6 | Words: Feigned > Correct |  |
|  |  |  | 4 | Words: Feigned > Incorrect |  |
|  |  |  | 5 | Words: Random > Correct |  |
| 29 | Mohamed et al., 2006 | 5 | 8 | (Lie, Known Lie + Lie, Subjective Lie) > Rest, Non-Guilty Subjects | Mock Shooting |
|  |  |  | 7 | (Truth, Known Truth + Truth, Subjective Truth) > Rest, Non-Guilty Subjects |  |
|  |  | 6 | 9 | Lie, Known Lie > Lie, Subjective Lie, Guilty Subjects |  |
|  |  |  | 7 | Truth, Subjective Truth > Truth, Known Truth, Guilty Subjects |  |
| 30 | Nunez et al., 2005 | 20 | 8 | False > True | True or False Response to Yes/No Questions |
|  |  |  | 7 | False, Autobiographical > True, Autobiographical |  |
|  |  |  | 4 | Autobiographical > Non-Autobiographical |  |
| 31 | Ofen et al., 2017 | 18 | 7 | Conjunction Analysis: Lie > True, Episodic and Belief | Lying (about personal experiences or beliefs) |
|  |  |  | 6 | Deception Main Effects: Belief-Lie > Belief-True & Episodic-Lie > Episodic-True |  |
|  |  |  | 13 | Preparation-Lie > Preparation-True |  |
|  |  |  | 11 | Negative Correlation between Preparation-Lie > Preparation-True and Deception Index |  |
| 32 | Peth et al., 2015 | 20 | 10 | Guilty Action > Neutral | Concealed Information Test |
|  |  |  | 1 | Guilty Intention > Neutral |  |
|  |  |  | 3 | Innocent > Neutral |  |
| 33 | Phan et al., 2005 | 14 | 11 | Lie > Truth | Modified Guilty Knowledge Test |
|  |  |  | 8 | Lie > Recognition |  |
| 34 | Pornpattananangkul et al., 2018 | 31 | 5 | Opportunity > No-Opportunity (covariate: Overall Dishonesty) | Modified Coin-guessing Task |
|  |  |  | 4 | Opportunity-Self > No-Opportunity-Self (covariate: Opportunity-Self Dishonesty) |  |
|  |  |  | 7 | Opportunity-Donation > No-Opportunity-Donation (covariate: Opportunity-Donation Dishonesty) |  |
|  |  |  | 4 | Opportunity-Self > Opportunity-Donation (covariate: Self Serving Dishonesty) |  |
|  |  |  | 7 | Opportunity > No-Opportunity |  |
|  |  |  | 2 | Opportunity-Self > Opportunity-Donation |  |
|  |  |  | 4 | Opportunity-Donation > Opportunity-Self |  |
|  |  |  | 2 | No-Opportunity > Opportunity (covariate: Overall Dishonesty) |  |
|  |  |  | 1 | No-Opportunity-Self > Opportunity-Self (covariate: Opportunity-Self Dishonesty) |  |
|  |  |  | 2 | Self > Donation (covariate: Self Serving Dishonesty) |  |
|  |  |  | 1 | No-Opportunity > Opportunity |  |
|  |  |  | 4 | Self > Donation |  |
|  |  |  | 4 | Donation > Self |  |
|  |  |  | 2 | No-Opportunity-Self > No-Opportunity-Donation |  |
|  |  |  | 6 | No-Opportunity-Donation > No-Opportunity-Self |  |
| 35 | Shao et al., 2017 | 48 | 3 | Dishonest (D) > Truthful (T); Cue Phase | Modified Directed Lie Paradigm |
|  |  | 23 | 1 | Low (L) > High (H) Psychopathic Personality Inventory (H), Dishonest > Truthful; Cue Phase |  |
|  |  | 48 | 10 | Initial Session (T1) > Testing Session (T2), Dishonest > Truthful; Cue Phase |  |
|  |  | 23 | 8 | (L(T2(D>T)>T1(D>T)) > H(T2(D>T)>T1(D>T)); Cue Phase |  |
|  |  | 48 | 5 | Dishonest > Truthful; Face-Responding Phase |  |
|  |  |  | 4 | Initial Session(Dishonest > Truthful) > Testing Session(Dishonest > Truthful); Face-Responding Phase |  |
|  |  | 23 | 3 | L(T2(D>T) > T1(D>T)) > H(T2(D>T) > T1(D>T))); Face-Responding |  |
|  |  |  | 2 | Low (Familiar > Unfamiliar) > High Psychopathic Personality Inventory (Familiar > Unfamiliar) |  |
|  |  | 48 | 10 | Initial Session > Testing Session; Control Visuo-spatial Control Task |  |
|  |  |  | 5 | Familiar > Unfamiliar |  |
|  |  |  | 1 | Unfamiliar > Familiar |  |
|  |  |  | 1 | Testing Session > Initial Session; Control Visuo-spatial Control Task |  |
|  |  | 23 | 1 | H(Testing Session-Initial Session) > L(Testing Session-Initial Session); Control Visuo-spatial Control Task |  |
|  |  |  | 1 | Low-High Psychopathic Personality Inventory; Control Visuo-spatial Control Task |  |
| 36 | Spence et al., 2008 | 17 | 7 | Lie - Truth | Decision-making (whether or not to lie) |
|  |  |  | 11 | [(Lie - Truth) - (Defy - Comply)] |  |
|  |  |  | 6 | Comply - Defy |  |
| 37 | Suchotzki et al., 2015 | 32 | 2 | Main effect of Proportion (1:1 > 1:4) | Mock Crime/Concealed Information Test |
| 38 | D. Sun et al., 2015b | 17 | 5 | Main effect of Response Type (Lie > (Truth) | Face Familiarity/Directed Lying |
|  |  |  | 1 | Interaction effect between Response Type and Face (Familiar(Lie-Truth) > Unfamiliar(Lie-Truth)) |  |
|  |  |  | 1 | Main effect of Face (Unfamiliar > Familiar) |  |
|  |  |  | 5 | Main effect of Face (Familiar>Unfamiliar) |  |
| 39 | D. Sun et al., 2015a | 25 | 5 | Dishonest > Honest (Positive Effect) | Economic Game |
|  |  |  | 2 | Dishonest > Honest (Negative Effect) |  |
|  |  |  | 4 | Main Effect of Detection (Not Detected > Detected) |  |
| 40 | D. Sun et al., 2016 | 25 | 6 | Dishonest > Honest | Economic Game |
|  |  |  | 1 | Computer (Dishonest-Honest) > Human (Dishonest-Honest) |  |
|  |  |  | 6 | Human > Computer |  |
| 41 | P. Sun et al., 2017 | 21 | 4 | Main Effects of Decision (Lying > Honest) | Adapted Dictator Game (after Ball-guess Game) |
|  |  |  | 1 | Interaction between Financial Position & Decision (Lying - Honest)Non-Deprived > (Lying-Honest)Deprived |  |
| 42 | Vartanian et al., 2012 | 15 | 7 | Lying > Truthful | Match/Mismatch Detection |
|  |  |  | 11 | Matched: Lying > Truthful |  |
|  |  |  | 5 | Mismatched: Lying > Truthful |  |
|  |  |  | 2 | Mismatched > Matched |  |
| 43 | Wu et al., 2011 | 20 | 8 | Bad Lie > Bad Truth | Evaluating Cultural Aspects of Lying |
|  |  |  | 6 | Bad Lie > Good Lie |  |
|  |  |  | 2 | Good Lie > Bad Lie |  |
| 44 | Yin et al., 2016a | 44 | 13 | Spontaneous Lie in Incorrect Prediction, Spontaneous Truth in Incorrect Prediction, Spontaneous Truth in Correct Prediction > Fixation | Modified Sic Bo Gambling |
| 45 | Yin & Weber, 2016b | 38 | 4 | Main Effect of Means (Lies > Truth) | Modified Cheap Talk Sender/Receiver Game |
|  |  |  | 2 | Main Effect of Means (Truth > Lies) |  |
|  |  |  | 2 | Main Effect of Ends (Beneficial > Harmful Outcome) |  |
| 46 | Yin et al., 2019 | 37 | 3 | Lying > Truth-telling | Color Reporting Game |
|  |  |  | 2 | Truth-telling > Lying |  |
|  |  |  | 12 | Negative Correlation between Lying and Lying Percentages |  |
|  |  |  | 6 | Negative Correlation between Lying > Truth-telling and Lying Percentages |  |
