# Supplementary material for: Meta-analytic connectivity modelling of deception-related brain regions
Source: PLoS One. 2021 Aug 25;16(8):e0248909. doi: 10.1371/journal.pone.0248909 (PMC8386837; doi:10.1371/journal.pone.0248909)
Supplement: S2 Table — Contrasts included Deceptive > Honest, Honest > Deceptive, etc. (DOCX) [file pone.0248909.s005.docx]

| **Cluster #** | **x** | **y** | **z** | **ALE** | **P** | **Z** | **Label (Nearest Gray Matter within 5mm)** | | |  |  |  |  |
| --- | --- | --- | --- | --- | --- | --- | --- | --- | --- | --- | --- | --- | --- |
| **1** | *-34* | *24* | *0* | *0.06885* | *4.13E-14* | *7.466457* | *Left Insula (BA 13)* |  |  |  |  |  |  |
|  | -34 | 22 | -8 | 0.065777 | 2.77E-13 | 7.211636 | Left Inferior Frontal Gyrus (BA 47) | | |  |  |  |  |
|  | -50 | 20 | -2 | 0.045171 | 3.96E-08 | 5.369113 | Left Inferior Frontal Gyrus (BA 47) | | |  |  |  |  |
|  | -50 | 18 | 12 | 0.043065 | 1.21E-07 | 5.164097 | Left Inferior Frontal Gyrus (BA 44) | | |  |  |  |  |
|  | -50 | 18 | 6 | 0.042346 | 1.75E-07 | 5.094211 | Left Inferior Frontal Gyrus (BA 44) | | |  |  |  |  |
|  | -50 | 14 | 22 | 0.038654 | 1.16E-06 | 4.722516 | Left Inferior Frontal Gyrus (BA 9) | | |  |  |  |  |
|  | -40 | 12 | 46 | 0.037749 | 1.83E-06 | 4.630052 | Left Middle Frontal Gyrus (BA 6) | | |  |  |  |  |
|  | -44 | 30 | -12 | 0.035902 | 4.56E-06 | 4.437222 | Left Inferior Frontal Gyrus (BA 47) | | |  |  |  |  |
|  | -42 | 10 | 24 | 0.03552 | 5.48E-06 | 4.397123 | Left Inferior Frontal Gyrus (BA 9) | | |  |  |  |  |
|  | -42 | 18 | 38 | 0.033429 | 1.50E-05 | 4.173541 | Left Middle Frontal Gyrus (BA 9) | | |  |  |  |  |
|  | -40 | 26 | 30 | 0.031055 | 4.55E-05 | 3.913247 | Left Middle Frontal Gyrus (BA 9) | | |  |  |  |  |
| **2** | *-4* | *18* | *50* | *0.072195* | *4.99E-15* | *7.739939* | *Left Superior Frontal Gyrus (BA 6)* | | |  |  |  |  |
|  | 8 | 12 | 60 | 0.045936 | 2.62E-08 | 5.443066 | Right Superior Frontal Gyrus (BA 6) | | |  |  |  |  |
|  | 8 | 20 | 38 | 0.025497 | 5.39E-04 | 3.26929 | Right Cingulate Gyrus (BA 32) | |  |  |  |  |  |
| **3** | *-58* | *-50* | *32* | *0.057895* | *3.16E-11* | *6.536025* | *Left Supramarginal Gyrus (BA 40)* | | |  |  |  |  |
|  | -44 | -46 | 44 | 0.050948 | 1.68E-09 | 5.912953 | Left Inferior Parietal Lobule (BA 40) | | |  |  |  |  |
|  | -34 | -58 | 44 | 0.036293 | 3.76E-06 | 4.47818 | Left Angular Gyrus (BA 39) | |  |  |  |  |  |
|  | -56 | -62 | 26 | 0.025474 | 5.46E-04 | 3.265673 | Left Superior Temporal Gyrus (BA 39) | | |  |  |  |  |
| **4** | *52* | *-46* | *40* | *0.069782* | *2.30E-14* | *7.542881* | *Right Inferior Parietal Lobule (BA 40)* | | |  |  |  |  |
|  | 42 | -44 | 36 | 0.053169 | 4.82E-10 | 6.115511 | Right Supramarginal Gyrus (BA 40) | | |  |  |  |  |
|  | 40 | -52 | 46 | 0.040636 | 4.24E-07 | 4.924064 | Right Inferior Parietal Lobule (BA 40) | | |  |  |  |  |
| **5** | *34* | *24* | *-4* | *0.073801* | *1.79E-15* | *7.868937* | *Right Insula* |  |  |  |  |  |  |
|  | 52 | 16 | -12 | 0.030482 | 5.95E-05 | 3.848378 | Right Inferior Frontal Gyrus (BA 47) | | |  |  |  |  |
|  | 50 | 22 | -16 | 0.02704 | 2.77E-04 | 3.452849 | Right Inferior Frontal Gyrus (BA 47) | | |  |  |  |  |
| **6** | *48* | *24* | *30* | *0.043454* | *9.85E-08* | *5.202246* | *Right Middle Frontal Gyrus (BA 9)* | | |  |  |  |  |
