# Supplementary material for: Meta-analytic connectivity modelling of deception-related brain regions
Source: PLoS One. 2021 Aug 25;16(8):e0248909. doi: 10.1371/journal.pone.0248909 (PMC8386837; doi:10.1371/journal.pone.0248909)
Supplement: S3 Table — (DOCX) [file pone.0248909.s006.docx]

| **Node Label** | **Papers** | **Subjects** | **Experiments** | **Conditions** | **Locations** |
| --- | --- | --- | --- | --- | --- |
| **1- L Ins** | 242 | 4092 | 321 | 730 | 5725 |
| **2- L SFG** | 200 | 3509 | 249 | 576 | 3851 |
| **3- R Ins** | 173 | 2898 | 217 | 458 | 3975 |
| **4- R SMG** | 71 | 1254 | 77 | 193 | 1057 |
| **5- L SMG** | 55 | 1111 | 62 | 137 | 959 |
| **6- L MFG** | 57 | 1003 | 63 | 138 | 908 |
| **7- R MFG** | 87 | 1629 | 99 | 217 | 1600 |
