# Supplementary material for: Meta-analytic connectivity modelling of deception-related brain regions
Source: PLoS One. 2021 Aug 25;16(8):e0248909. doi: 10.1371/journal.pone.0248909 (PMC8386837; doi:10.1371/journal.pone.0248909)
Supplement: S4 Table — Using “Paradigm Analysis” plugin for Mango [32]. Z-scores that are significant according to Lancaster et al. [32], meaning they have a z-score of > = 3.0, are in bold. However, all z-scores > = 2.0 are reported here. (DOCX) [file pone.0248909.s007.docx]

|  | **Regions** | | | | |
| --- | --- | --- | --- | --- | --- |
|  | **L Ins** | **L SFG** | **R Ins** | **R SMG** | **R MFG** |
| **Affective Pictures** | 2.6721 | - | - | - | - |
| **Counting/Calculation** | **3.6978** | **3.1098** | 2.4121 | - | - |
| **Cued Explicit Recognition/Recall** | **3.6366** | 2.5466 | - | - | 2.5593 |
| **Delayed Match to Sample** | 2.6302 | **3.9447** | - | - | - |
| **Encoding** | - | **3.0168** | - | - | - |
| **Face Monitor/Discrimination** | 2.8388 | - | - | - | - |
| **Finger Tapping/Button Press** | **3.4896** | **4.9046** | **3.7170** | - | - |
| **Go/No-Go** | **3.9525** | **3.1135** | **3.8162** | - | 2.0411 |
| **Imagined Objects/Scenes** | 2.1082 | - | - | - | - |
| **Mental Rotation** | - | 2.1117 | - | - | - |
| **n-back** | **3.3162** | **3.4507** | 2.2872 | 2.8463 | - |
| **Naming (Covert)** | 2.6537 | - | - | - | - |
| **Oddball Discrimination** | - | - | 2.0191 | - | - |
| **Pain Monitor/Discrimination** | **3.4691** | 2.3228 | **5.5500** | - | - |
| **Paired Associate Recall** | 2.2815 | 2.6731 | - | - | - |
| **Phonological Discrimination** | 2.7953 | 2.9610 | - | - | - |
| **Reading (Covert)** | 2.3430 | 2.1214 | - | - | - |
| **Reading (Overt)** | 2.0357 | - | - | - | - |
| **Reasoning/Problem Solving** | - | 2.1704 | - | - | - |
| **Recitation/Repetition (Overt)** | 2.5649 | - | - | - | - |
| **Reward** | **4.5640** | 2.4270 | **4.9591** | - | - |
| **Semantic Monitor/Discrimination** | **3.0389** | **4.3118** | 2.3657 | - | - |
| **Stroop-Color** | - | 2.5754 | - | - | - |
| **Tactile Monitor/Discrimination** | - | 2.0993 | - | - | - |
| **Task Switching** | - | **3.3788** | - | - | - |
| **Taste** | 2.6531 | - | 2.4513 | - | - |
| **Tone Monitor/Discrimination** | 2.6278 | **3.2935** | - | - | - |
| **Visual Object Identification** | 2.1451 | - | - | - | - |
| **Visual Pursuit/Tracking** | - | 2.5556 | - | - | - |
| **Visuospatial Attention** | **3.8377** | 2.8203 | 2.6514 | - | - |
| **Word Generation (Covert)** | **3.2692** | 2.9425 | - | - | - |
| **Word Generation (Overt)** | 2.7070 | 2.5111 | - | - | - |
