# Supplementary material for: Meta-analytic connectivity modelling of deception-related brain regions
Source: PLoS One. 2021 Aug 25;16(8):e0248909. doi: 10.1371/journal.pone.0248909 (PMC8386837; doi:10.1371/journal.pone.0248909)
Supplement: S5 Table — Using “Behavioral Analysis” plugin for Mango [32]. Z-scores that are significant according to Lancaster et al. [32], meaning they have a z-score of > = 3.0, are in bold. However, all z-scores > = 2.0 are reported here. (DOCX) [file pone.0248909.s008.docx]

|  |  | **Regions** | | | | | |
| --- | --- | --- | --- | --- | --- | --- | --- |
|  |  | **L Ins** | **L SFG** | **R Ins** | **R SMG** | **L SMG** | **R MFG** |
| *Action* | **Execution (Speech)** | **3.0081** | 2.0774 | - | - | - | - |
|  | **Execution (Unspecified)** | 2.7923 | 2.6188 | - | - | - | - |
|  | **Inhibition** | **3.4251** | 2.7962 | **3.5550** | 2.0475 | - | 2.0475 |
| *Cognition* | **Attention** | **5.8371** | **6.7798** | **6.4210** | 2.3341 | - | **3.1239** |
|  | **Language (Orthography)** | 2.1321 | - | - | - | - | - |
|  | **Language (Phonology)** | **3.2077** | **3.7727** | - | - | - | - |
|  | **Language (Semantics)** | **6.0369** | **5.3346** | **3.2717** | - | - | - |
|  | **Language (Speech)** | **6.0968** | **4.5178** | **3.1895** | - | - | - |
|  | **Memory (Explicit)** | **4.8853** | **4.0074** | **3.2422** | - | - | 2.6640 |
|  | **Memory (Working)** | **4.5278** | **5.8289** | 2.0587 | 2.4350 | - | - |
|  | **Music** | 2.7857 | **3.2495** | - | - | - | - |
|  | **Reasoning** | **5.6930** | **4.4441** | **3.3908** | - | - | - |
|  | **Social Cognition** | - | 2.4984 | - | - | 2.5222 | - |
|  | **Spatial** | 2.3524 | 2.1314 | 2.5416 | - | - | - |
| *Emotion* | **Negative (Disgust)** | 2.1034 | - | - | - | - | - |
|  | **Negative (Fear)** | - | - | 2.1721 | - | - | - |
|  | **Negative (Sadness)** | 2.2809 | - | - | - | - | - |
|  | **Negative (Unspecified)** | - | 2.2410 | - | - | - | - |
|  | **Positive (Reward/Gain)** | **4.2764** | 2.5085 | **4.6898** | - | - | - |
| *Interoception* | **Thermoregulation** | 2.6764 | - | **3.0325** | - | - | - |
| *Perception* | **Audition** | **3.6733** | **4.1591** | 2.2410 | - | - | - |
|  | **Gustation** | 2.7197 | - | 2.7146 | - | - | - |
|  | **Somesthesis (Pain)** | **3.8175** | 2.2596 | **5.4171** | - | - | - |
|  | **Somesthesis (Unspecified)** | - | 2.5134 | - | - | - | - |
|  | **Vision (Color)** | - | 2.2056 | - | - | - | - |
|  | **Vision (Motion)** | 2.0608 | 2.6348 | - | - | - | - |
|  | **Vision (Shape)** | **3.3639** | 2.7276 | - | - | - | - |
|  | **Vision (Unspecified)** | 2.3573 | 2.5158 | - | - | - | - |
